# Effective Heart Disease Detection Based on Quantitative Computerized Traditional Chinese Medicine Using Representation Based Classifiers

**DOI:** 10.1155/2017/7483639

**Published:** 2017-08-13

**Authors:** Ting Shu, Bob Zhang, Yuan Yan Tang

**Affiliations:** Department of Computer and Information Science, University of Macau, Taipa, Macau

## Abstract

At present, heart disease is the number one cause of death worldwide. Traditionally, heart disease is commonly detected using blood tests, electrocardiogram, cardiac computerized tomography scan, cardiac magnetic resonance imaging, and so on. However, these traditional diagnostic methods are time consuming and/or invasive. In this paper, we propose an effective noninvasive computerized method based on facial images to quantitatively detect heart disease. Specifically, facial key block color features are extracted from facial images and analyzed using the Probabilistic Collaborative Representation Based Classifier. The idea of facial key block color analysis is founded in Traditional Chinese Medicine. A new dataset consisting of 581 heart disease and 581 healthy samples was experimented by the proposed method. In order to optimize the Probabilistic Collaborative Representation Based Classifier, an analysis of its parameters was performed. According to the experimental results, the proposed method obtains the highest accuracy compared with other classifiers and is proven to be effective at heart disease detection.

## 1. Introduction

Heart disease (HD) is actually a broad term used for a wide variety of diseases of the heart and blood vessels such as coronary artery disease (CAD) [1] and heart rhythm disorders called arrhythmias (ARR) [2]. According to the World Health Organization (WHO), HD is the number one cause of death globally [3]. In 2012, it was estimated that HD caused about 17.5 million deaths, which means a person died from HD every 2 seconds [4]. There are many tests to diagnose HD; the main traditional diagnostic methods of HD are [5] blood tests, Electrocardiogram (ECG) [6], Holter monitoring [7], echocardiogram [8], cardiac catheterization [9], cardiac computerized tomography (CT) scan [10], and cardiac magnetic resonance imaging (MRI) [11].

Many clues about the health of a person's heart can be discovered in his/her blood. However, a single blood test cannot reflect the risk of heart disease. Two common blood tests for heart disease are a cholesterol test and a C-reactive protein (CRP) test. These tests analyze cholesterol and CRP contents in the blood, respectively, while overall the results can help create a clear picture of a person's heart health [12]. An ECG records electrical signals, while a Holter monitor is a portable device the patient wears to record a continuous ECG, usually for 24 to 72 hours. An echocardiogram uses sound waves to produce images of a person's heart, while a stress test records a person's signs and symptoms during exercise using an ECG or echocardiogram. For cardiac catheterization, a special dye needs to be injected into a person's coronary arteries through a long, thin, and flexible tube (catheter) usually in the leg. The dye then outlines narrow spots and blockages that appear in X-ray images. A CT scan and MRI can also help doctors detect calcium deposits in the patient's arteries that can narrow it.

Blood tests performed on individuals with HD are considered invasive as bodily fluids are removed and can take time for the laboratory technician to reach a result. ECG on the other hand might not be as invasive as a blood test, but in the case of Holter monitoring, it is time consuming. As for cardiac catheterization, the injection of a special dye is the definition of invasive. Therefore, given these issues, there is a need to develop a noninvasive computerized method to detect HD.

In 2008, Kim et al. proposed one such method to conduct the color compensation of a facial image based on the analysis of facial color [13] rooted in Traditional Chinese Medicine (TCM). In [13], they extracted the center forehead and lips of a person and analyzed the red color value distribution of the center forehead and lips. The authors wanted to survey real clinical data of HD patients and group them into different cases based on the analysis that facial color can help doctors diagnose HD. However, the authors just proposed a method and did not experiment on a real dataset.

Recently, Zhang et al. [14] used facial block color features to detect diabetes in a noninvasive manner with the Sparse Representation Based Classifier (SRC). Even though their detection results are relatively high, further analyses using other representation algorithms have not been studied nor have these algorithms been applied to detect other nondiabetic diseases. To resolve these issues, we propose an effective noninvasive computerized method to detect HD through facial image analysis via the Probabilistic Collaborative Representation Based Classifier (ProCRC) and apply our proposed method on a real dataset. ProCRC was first proposed in [15] and applied in pattern recognition, being developed from the Collaborative Representation Based Classifier (CRC) of [16]. Zhang et al. [16] proved that Collaborative Representation played a more important role than sparsity in pattern recognition and proposed CRC, which outperformed the SRC [17] and also runs much faster. In our work, the ProCRC was modified to be applied for HD detection based on facial key block color features. The ProCRC combines CRC and the probabilistic theory.

For the proposed method, facial images are first captured through a specially designed facial image capture device and four facial key blocks are extracted from each image. A color gamut with six-facial-color centroids is employed to extract color features from each block. The dataset used in this paper has two distinctive classes: (1) HD with 581 samples and (2) healthy (H) consisting of 581 samples. Based on the seven facial key block permutations, ProCRC with its optimal parameters is applied to classify HD versus H. To the best of our knowledge, this is the first time noninvasive computerized heart disease detection has been proposed in the literature.

The organization of this paper is given as follows. The details about the dataset are represented in Section 2. Feature extraction of the facial key blocks is given in Section 3, succeeded by a description of our proposed method in Section 4 using ProCRC.  Section 5 describes and discusses the experimental results and Section 6 concludes this paper.

## 2. Dataset

The dataset we collected and used in this work consists of 581 H and 581 HD samples from the Guangdong Provincial TCM Hospital, Guangdong, China, in 2015. Individuals were diagnosed as healthy by medical professional practicing Western medicine, while heart disease patients were determined using the methods described in Section 1. Please note the handling of human subjects was done according to the principles outlined in the Declaration of Helsinki and each individual gave their consent to be a part of this study. Ethical approval was obtained from the Science and Technology Development Fund (FDCT) of Macao for this study with the project number FDCT 124/2014/A3.

**Figure 1:**
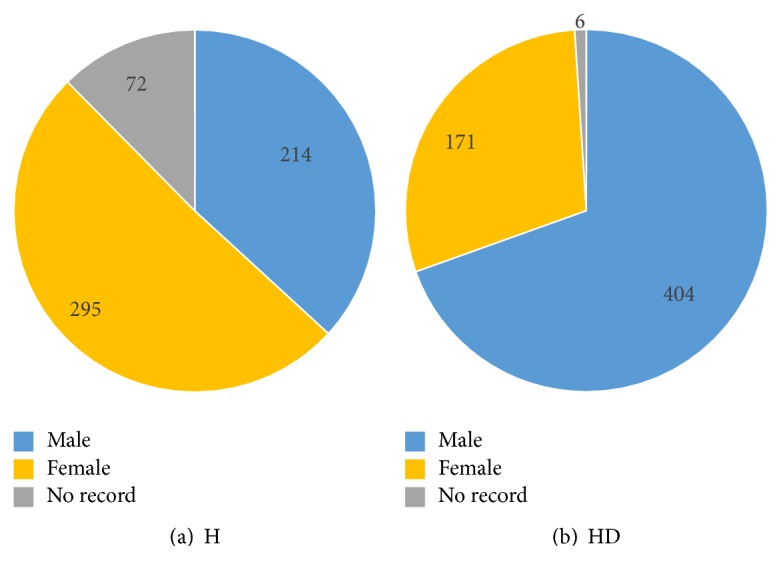
Gender distribution of the dataset.

**Figure 2:**
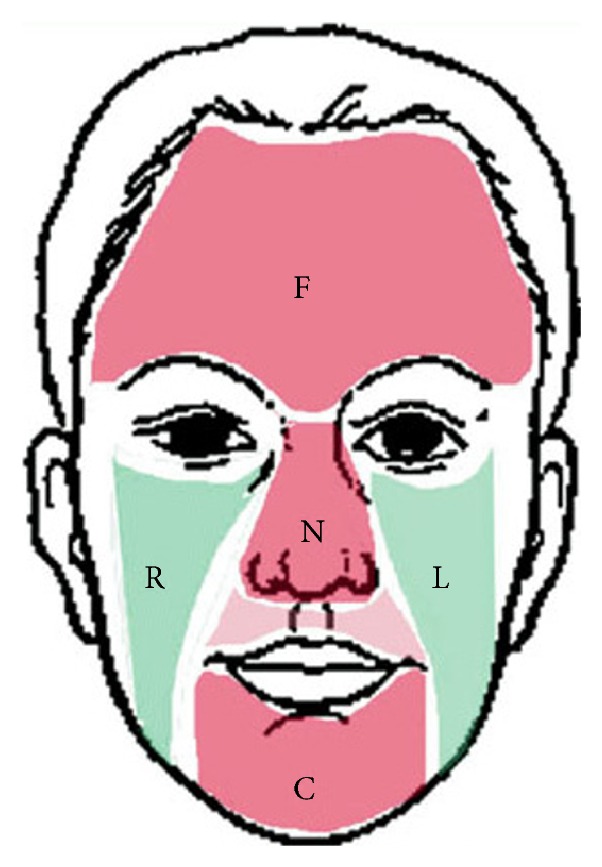
Different facial regions according to TCM.

The gender and age distributions of H and HD are described in this section. During data collection, it is sometimes difficult to record the information of everyone due to many circumstances. Therefore, in gender and age distributions, there are cases of no record (NR). The following pie charts (Figure 1) are used to show the dataset gender distribution. In the pie chart, blue represents males, yellow is for females, and NR is illustrated in gray. In Figure 1, there are two pie charts describing the gender distributions of the dataset: (1) H (Figure 1(a)) and (2) HD (Figure 1(b)). According to Figure 1(a), 72 people are missing their gender information in H and about half of the healthy dataset is female (295), while the number of males is 214. Different from the H dataset, the HD dataset has only 6 NR cases. About 1/3 of the HD patients are female (171) with 404 male HD patients 2/3 (see Figure 1(b)).

The age distribution is given through a table (see Table 1). To show the age distribution (in years) clearly, the age is split into 5 parts: [1–17], [18–24], [25–60], [61–80], and [≥81]. From this table, the first column is the class name, where each class has two rows: the first row is the number of the people belonging to the age range and the second row is the corresponding percentage of people out of the total. For the H dataset, the age of most people is from 18 to 60 (56.28% + 30.81% = 87.09%) with no healthy person above 80 and it contains only 4 people above 60. As for the HD dataset consisting of 581 samples, the majority of HD patients are aged from 25 to 80 (68.5% + 19.79% = 88.29%).

It should be noted that the missing gender and age information does not affect our study since we are only interested in each individual's health status.

## 3. Facial Key Block Feature Extraction

In order to decrease the effects of the capture environment, a specially designed facial image capture device was applied. Using the device, the individual just needs to place his/her head on the chin rest and the device operator clicks the capture button. More details about the device can be found in [14]. A color correction procedure [18] was also performed to portray the facial images in an accurate way after image capture.

In Traditional Chinese Medicine (TCM), it is believed that the status of the internal organs can be determined from different regions of the face [19–21].  Figure 2 shows a human face partitioned into various regions according to TCM [22]. Facial blocks were previously defined in [23] to detect hepatitis from digital facial images. The authors extracted 5 facial blocks, one between the eyebrows, two below the eyes, one under the bridge of the nose, and one underneath the lower lip. Applying this idea to our proposed method, four facial key blocks are automatically extracted from each facial image representing the main regions. No facial block is used to represent region C in Figure 2 due to the existence of facial hair.

**Figure 3:**
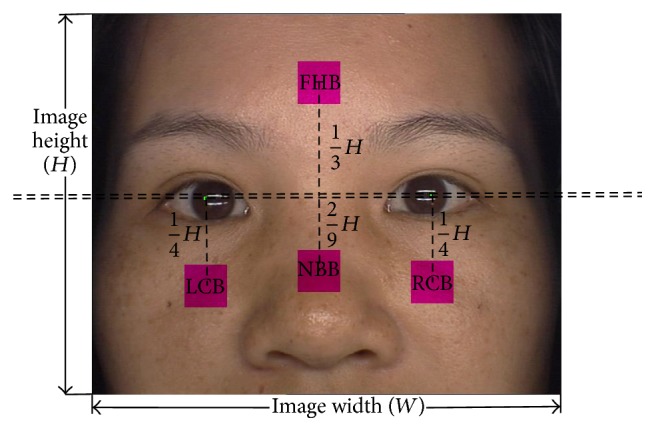
Four facial key block positions.

Hence, according to the five facial regions, four facial key blocks are automatically extracted from each calibrated facial image. Furthermore, the dimensionality of the whole facial image is much larger than four facial key blocks. Therefore, using four facial key blocks instead of the whole facial image is more appropriate and efficient.  Figure 3 depicts an example of a facial image with its four marked facial key blocks. The four facial key blocks are forehead block (FHB) on the forehead, left and right cheek blocks (LCB and RCB) below the left and right eyes which are symmetrical, and nose bridge block (NBB) on the nose, the midpoint of LCB and RCB. The four facial key block sizes are the same at 64 × 64 pixels.

In the automatic key blocks extraction procedure, the pupils are first detected and marked. The positions of the two pupils are denoted as *L*_*lp*_ = (*x*_*lp*_, *y*_*lp*_) (left) and *L*_*rp*_ = (*x*_*rp*_, *y*_*rp*_) (right). Based on *L*_*lp*_ and *L*_*rp*_, the four facial key blocks are located through

**Figure 4:**
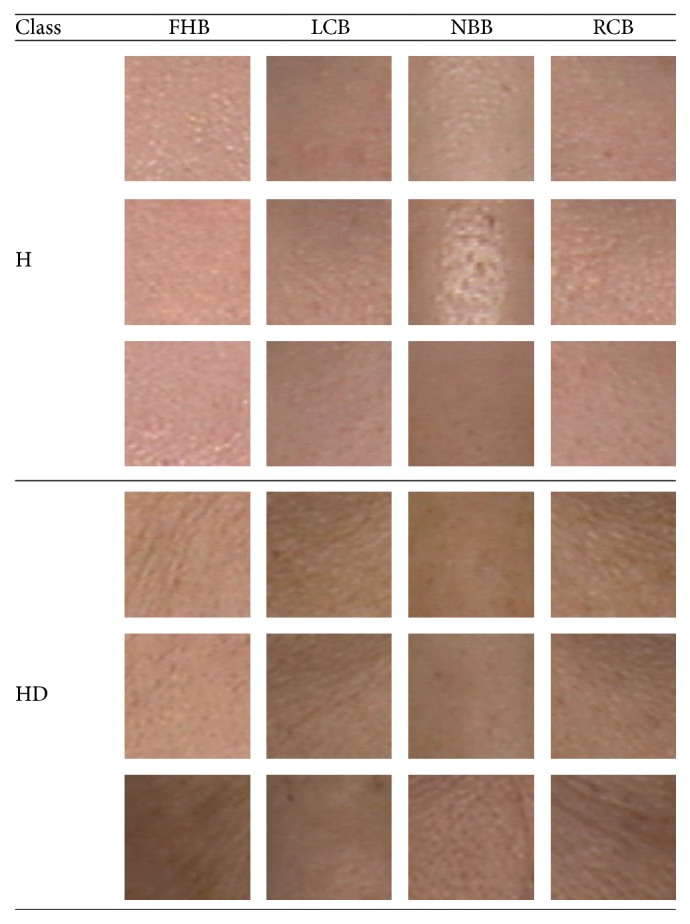
Three typical examples of four facial key blocks from the two classes.

(1) LLCB=xlp,ylp−14H,LRCB=xrp,yrp−14H,LFHB=xlp+xrp2,ylp+yrp2+13H,LNBB=xlp+xrp2,ylp+yrp2−29H,

where *L*_*i*th  key  block  name_ means the position of *i*th key block, such as *L*_FHB_ is the position of FHB, and *W* and *H* are the width and height of the facial image, respectively.  Figure 3 depicts the locations of the four facial key blocks based on the left and right pupil positions. Three typical examples from each class are illustrated in Figure 4.

**Figure 5:**
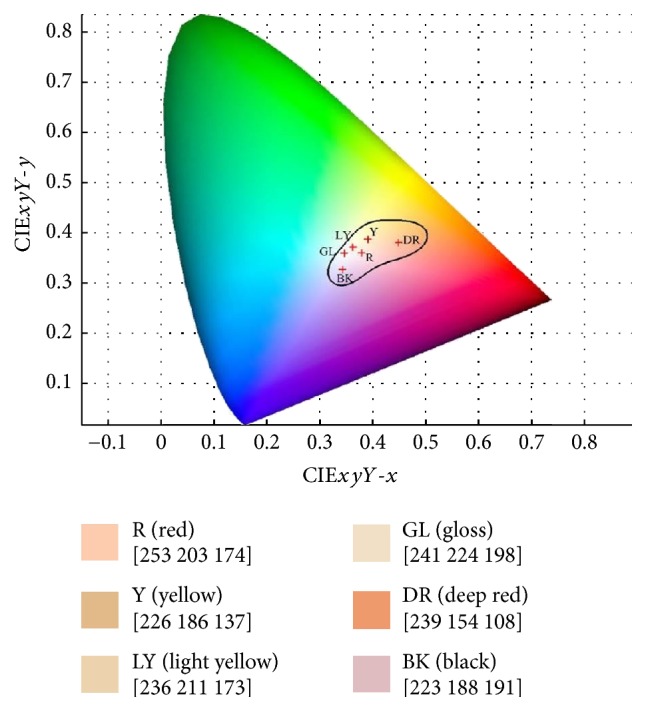
Facial color gamut with its six-color centroids marked by red crosses.

The color features are extracted from each facial key block. A color gamut (see Figure 5) with six-facial-color centroids are applied for color feature extraction, where 6 color values are extracted from each facial key block. Figure 5 illustrates the six-color centroids from the facial color gamut as a solid colored square, whose label is on top and correspondingly RGB value is below.

Each pixel in a facial block is compared to one of the six-color centroids and assigned to its nearest centroid. After evaluating all pixels of a facial block, the total of each color (based on the six-color centroids) is summed and divided by the total number of pixels. This ratio forms the facial color feature vector *k*, where *k* = [*r*_1_, *r*_2_, *r*_3_, *r*_4_, *r*_5_, *r*_6_] and *r*_*i*_ represents the sequence of the six-color centroids in Figure 5.

By comparing the four facial color feature vectors (per facial image) in groups of two (using all images in the dataset), and calculating the mean absolute difference of each group, LCB and RCB are shown to have the smallest difference [14]. This is not surprising given LCB and RCB are symmetrical and located on either side of the face. Therefore, in the following experiments, RCB is removed.

## 4. Representation Based Classifiers

### 4.1. Sparse Representation Based Classifier (SRC)

The SRC was first proposed by Wright et al. [17] and used for face recognition. Since then, this classifier has been applied in numerous fields such as pattern recognition [14, 24], object detection [25], image restoration [26], image denoising [27], video restoration [28], image super-resolution [29]. For the following, *D* represents a dataset; *s* donates a sample; *X*, *Y*, or *Z* stands for a coefficient; and *α* or *β* is a positive scalar.

The principle of the SRC is using the linear combination of the training data (*D*) to represent the query testing sample (*s*) while keeping the coefficients (*Y*) sparse enough. The coefficients of the class that the testing samples belong to have significant values, while the other coefficients are nearly zero. The SRC is defined as

(2) $$\begin{array}{c} \left(\;\hat{Y}\;\right)=\arg\underset{Y}{\min}\left\{\;{\left\|\;s-DY\;\right\|}_{2}^{2}+\alpha_{SRC}{\left\|\;Y\;\right\|}_{1}\;\right\}, \end{array}$$

where *α*_SRC_ can be set to obtain the real sparse coding vector $\hat{Y}$ of *s* over *D*.

### 4.2. Collaborative Representation Based Classifier (CRC)

In [16], Zhang et al. established the Collaborative Representation (CR) mechanism, but not the *l*_1_-norm sparsity constraint, that truly improved the method's effectiveness and further proposed a Collaborative Representation Based Classifier (CRC).

The authors of [16] proposed CRC by modifying the *l*_1_-norm of the SRC (2) to a *l*_2_-norm:

(3) $$\begin{array}{c} \left(\;\hat{Z}\;\right)=\arg\underset{Z}{\min}\left\{\;{\left\|\;s-DZ\;\right\|}_{2}^{2}+\alpha_{CRC}{\left\|\;Z\;\right\|}_{2}^{2}\;\right\}, \end{array}$$

where *α*_CRC_ is the regularization parameter. The solution of (3) can be easily and analytically derived as

(4) $$\begin{array}{c} \hat{Z}={\left(\;D^{T}D+\alpha_{CRC}\cdot I\;\right)}^{-1}D^{T}s. \end{array}$$

The first part ((*D*^*T*^*D* + *α*_CRC_ · *I*)^−1^*D*^*T*^) of (4) is independent of *s*. Therefore, it can be precalculated and once a query sample *s* is available, it is projected to get $\hat{Z}$. This makes calculating $\hat{Z}$ faster than $\hat{Y}$ in (2). More details about CRC can be found in [16].

### 4.3. Probabilistic Collaborative Representation Based Classifier (ProCRC)

Cai et al. [15] proposed the Probabilistic Collaborative Representation Based Classifier (ProCRC) algorithm for pattern classification. Let *D* = [*D*_1_, *D*_2_,…, *D*_*L*_] ∈ *ℝ*^*M*×*N*^ denote the training samples, where *D*_*l*_ ∈ *ℝ*^*M*×*N*_*l*_^ represents the training samples from the *l*_th_ class with *N*_*l*_ samples (*N* = ∑_*l*=1_^*L*^*N*_*l*_), and the dimension of each sample is *M*. The coefficient *X* of *D* representing a test sample *s* ∈ *ℝ*^*M*×1^ via ProCRC is solved with the following:

(5) X^=arg⁡minX⁡s−DX22+αX22+βL∑lLDX−DlXl22,

where *α* and *β* are regularization parameters.

Using ProCRC, the class label of the test sample is determined through locating the minimum value of the residual error for each class:

(6) $$\begin{array}{c} id\left(\;s\;\right)=\arg{\underset{l}{\min}\left\|D\hat{X}-D_{l}{\hat{X}}_{l}\right\|}_{2}^{2}, \end{array}$$

where ${\hat{X}}_{l}$ represents the coefficients of the test sample *s* in the *l*_th_ class.  Algorithm 1 shows the procedure of ProCRC. In order to show the ProCRC procedure clearly, let *D*_*l*_′ = [0,…, *D*_*l*_,…, 0] ∈ *ℝ*^*M*×*N*^ and ${\bar{D_{l}}}^{\prime }=D-D_{l}^{\prime }$ have the same size of *D*. More details about ProCRC can be found in [15].

## 5. Experimental Results

The experimental results are represented in this section. The settings for HD detection are first given followed by the detection results using 10 classifiers to compare and contrast with the ProCRC. Finally, the analysis of the ProCRC parameters *α* and *β* is represented in Section 5.3.

### 5.1. Experimental Setting

We randomly selected close to half (580) of the data for training and the remaining data (582) for testing, where accuracy (which is the proportion of the correctly classified samples divided by all samples) is the performance measurement used. To overcome the shortcoming of different results for different data partitions [30], 5 random partitions were applied, where the final accuracy is its mean. The following experimental results were conducted on a PC with 8 i7-6700 CPU @3.40 GHz processor, 16.0 GB RAM, and a 64-bit OS.

The dataset we collected and used in this work consists of 581 H and 581 HD samples from the Guangdong Provincial TCM Hospital, Guangdong, China, in 2015.

Based on Section 3, three facial key blocks (FHB, LCB, and NBB) are used instead of the whole facial image. Therefore, there are seven combinations for the three facial key blocks and all seven combinations were applied separately for each classifier. The seven block combinations consist of 3 cases with one block (FHB, LCB, and NBB), 3 cases with 2 blocks (FHB + LCB, FHB + NBB, and LCB + NBB), and all blocks combined together (FHB + LCB + NBB).

### 5.2. HD Detection Results

Other than the ProCRC, 10 other classifiers were applied to detect HD representing an array of traditional and the state of the art. The 10 classifiers are (i) *k*-Nearest Neighbor (*k*-NN) [31] with *k* = 1, (ii) Support Vector Machines (SVM) [31] with linear kernel function, (iii) SRC [17] with *λ* = 0.1, (iv) Dictionary Learning (DL) with SRC [32] using *λ*_SRC_ = 0.1, *λ*_DL_ = 0.1, and a dictionary size equal to half of the feature dimensionality, such as 3 for one key block, (v) CRC [16] with *λ* = 0.01, (vi) Softmax [33], (vii) Decision Tree [34], (viii) AdaBoost [35] with Tree Leaner, (ix) LogitBoost [36] with Tree Leaner, and (x) Gentle Boost [37]. The classifier parameters were fine-tuned based on its best performance and for the ProCRC its two parameters are analyzed in Section 5.3.

**Figure 6:**
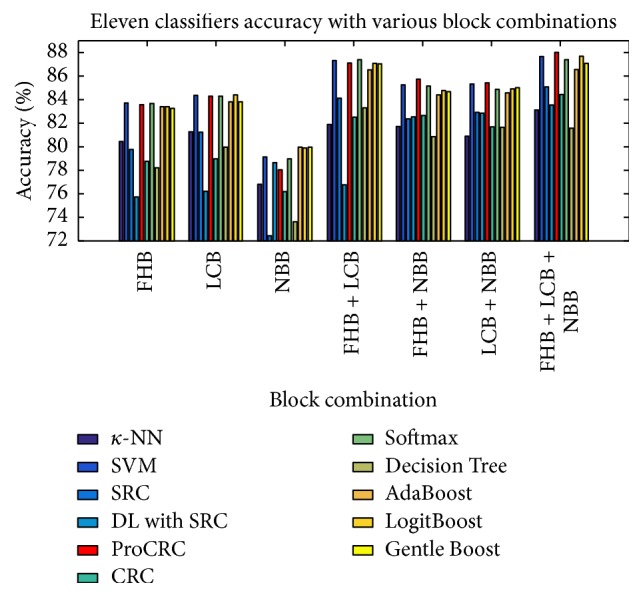
HD detection accuracies of all 11 classifiers including ProCRC.

Figure 6 illustrates the best accuracies of all 11 classifiers based on facial key block color features for all seven block combinations. From this bar chart, it is obvious that the ProCRC results (in red) outperformed or came close to achieving the highest accuracy for almost each combination.

To be thorough, the complete set of results including accuracy, sensitivity, and specificity [38] of the 11 classifiers using seven block combinations is shown in Table 2. In the table, ACC, SEN, and SPC represent accuracy, sensitivity, and specificity, respectively. As can be seen in Table 2, the ProCRC using FHB + LCB + NBB (highlighted) achieved the highest result (88.01%) amongst all classifiers. Using this grouping, the second highest result was 87.7% obtained by LogitBoost. The biggest difference between the ProCRC and the 10 other classifiers with FHB + LCB + NBB was 6.43%, where the classifier was Decision Tree. When compared to the representation based algorithms (SRC, DL with SRC, and CRC), the ProCRC achieved on average a 3.65% increase in accuracy using FHB + LCB + NBB.

**Figure 7:**
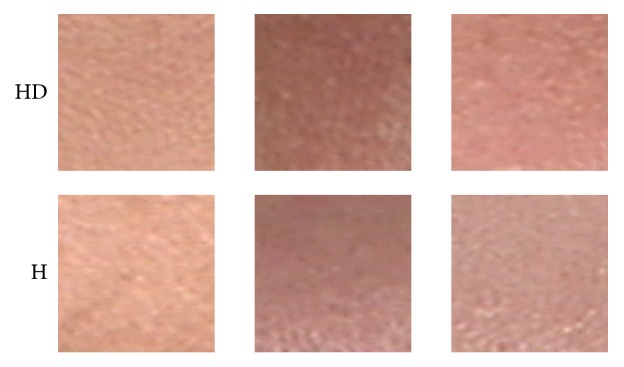
Three examples of FHB from HD and H that cannot be recognized with the naked eye.

To further demonstrate the effectiveness of the proposed method, Figure 7 shows three examples of FHB for HD and H, respectively. In this figure, the top row is FHB from HD and the bottom row is from H. Looking at the figure, it is difficult to distinguish the blocks with the naked eye. However, the proposed method can classify each block correctly.

### 5.3. ProCRC Parameters Analysis

**Figure 8:**
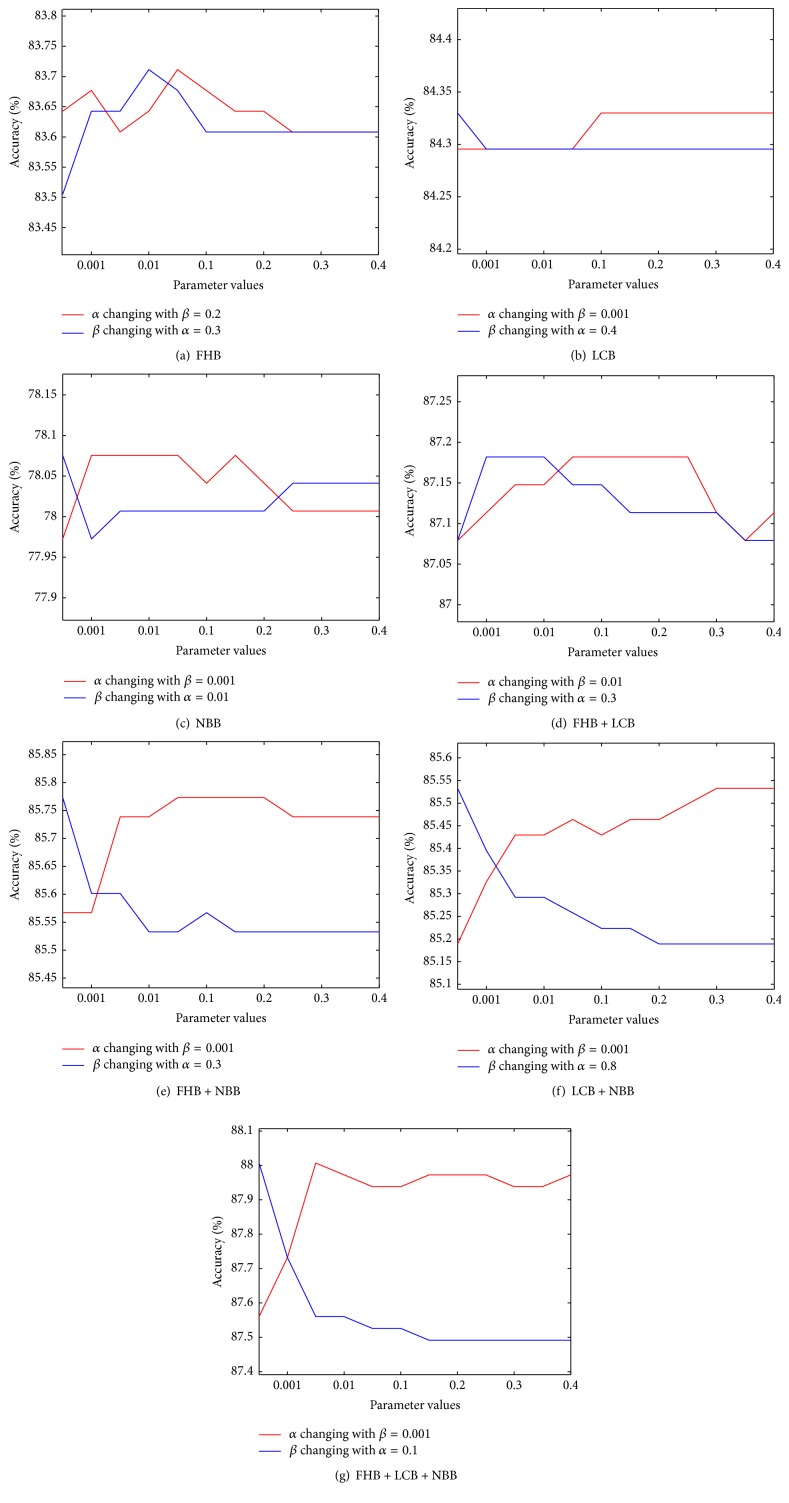
ProCRC accuracy with *α* and *β* changing.

Based on Section 4.3, both of the two parameters range from [0.001,0.01,0.1 : 0.1 : 1.0]. In order to find the optimal values of *α* and *β* for HD detection, experiments using each of the seven block combinations were analyzed. These results are shown in Figure 8. In each subfigure, the red line represents the accuracies of a fixed *β* with *α* changing its values, while in the blue line it is the opposite with *α* being equal to a constant and *β* changing.

*α* and *β* results based on FHB are shown in Figure 8(a). After *α* = 0.7 and *β* = 0.4, the red and blue lines remained constant, respectively. The best accuracy of FHB was 83.71%, where *α* = 0.3 and *β* = 0.2.  Figure 8(b) depicts the ProCRC parameter results for LCB. Except for *β* = 0.001, the accuracies of the other *β* values were the same. For *α*, the accuracies also had only two values, which were the same with *β*, where 0.4 caused a change. The ProCRC with *α* = 0.4 and *β* = 0.001 based on LCB obtained the best accuracy of 84.33%. The results for NBB are represented in Figure 8(c). For *β*, the top accuracy was achieved at the initial point (*β* = 0.001). The result of *α* from 0.01 to 0.3 did not change and the highest accuracy was 78.08%.  Figure 8(d) illustrates *α* and *β* for FHB + LCB. The best accuracies of *α* were the same (87.18%) from 0.3 to 0.7. The two parameters of ProCRC based on FHB + NBB are depicted in Figure 8(e). The *β* results decreased with the increasing of *β*. In contrast, the accuracies of *α* increased with the increasing of *α*. The highest accuracy of FHB + NBB was 85.77%, where *α* = 0.3 and *β* = 0.001.  Figure 8(f) shows the result of *α* and *β* for LCB + NBB. With the increasing of *α* and *β*, its accuracies increased and decreased, respectively. The best result of 85.53% was obtained from LCB + NBB with *α* = 0.8 and *β* = 0.001. The final subfigure (Figure 8(g)) represents the two parameters for FHB + LCB + NBB. Similar to Figure 8(f), the results decreased with an increasing *β*. From 0.001 to 0.1, the *α* accuracies increased with *α* increasing and had small fluctuations after 0.2. The best accuracy in this case, which was also the highest accuracy in all 11 classifier, was 88.01%, where *α* = 0.1 and *β* = 0.001.

## 6. Conclusions

This paper proposed a noninvasive computerized method to detect HD based on facial key block color analysis classified using the ProCRC. The experiments were conducted on a new dataset consisting of 581 HD samples and 581 H samples. The facial images are first captured through a specially designed device, where four facial key blocks are extracted to represent one sample. For each facial key block, color features are extracted using a facial color gamut with six-color centroids. To obtain optimal HD detection, three facial key blocks are permuted and applied for classification. The proposed method used the ProCRC which was developed from CRC and analyzed CRC based on the probabilistic theory [15]. Compared with 10 other classifiers, the best accuracy of HD detection was 88.01% with a sensitivity of 84.95% and a specificity of 91.07% (using the ProCRC with *α* = 0.1 and *β* = 0.001 with FHB + LCB + NBB). This proves the effectiveness of the ProCRC based on facial key block color feature analysis to detect HD and potentially provides a new innovative noninvasive way to detect this disease.

As part of the future work, more features from the facial key blocks will be explored and extracted. In addition, other representation learning algorithms will be developed and applied to HD detection.

## Figures and Tables

**Algorithm 1:**
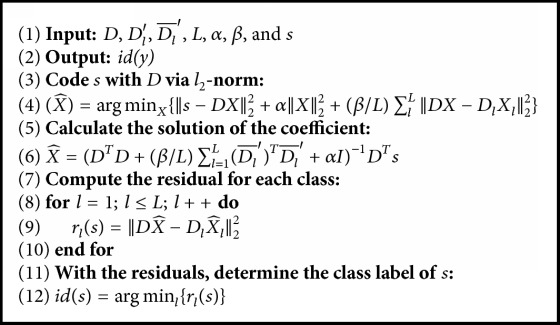
ProCRC algorithm procedure.

**Table 1:** Age distribution of the dataset.

Class	1–17	18–24	25–60	61–80	≥81	NR	Sum
H	1	327	179	4	0	70	581
	0.17%	56.28%	30.81%	0.69%	0%	12.05%	100%
HD	6	38	398	115	18	6	581
	1.03%	6.54%	68.5%	19.79%	3.1%	1.03%	100%

**Table 2:** Comprehensive HD detection results using 11 classifiers.

Block combination	ACC	SEN	SPC
	*k*-NN		

FHB	80.45%	67.56%	93.33%
LCB	81.27%	68.11%	94.43%
NBB	76.80%	63.99%	89.62%
FHB + LCB	81.89%	69.69%	94.09%
FHB + NBB	81.72%	71.27%	92.16%
LCB + NBB	80.89%	69.69%	92.10%
FHB + LCB + NBB	83.13%	72.30%	93.95%

	SVM		

FHB	83.71%	75.74%	91.68%
LCB	84.36%	76.01%	92.71%
NBB	79.11%	69.83%	88.38%
FHB + LCB	87.32%	82.47%	92.16%
FHB + NBB	85.26%	80.07%	90.45%
LCB + NBB	85.33%	78.28%	92.37%
FHB + LCB + NBB	87.66%	83.78%	91.55%

	SRC		

FHB	79.76%	77.18%	82.34%
LCB	81.24%	77.73%	84.74%
NBB	72.44%	69.62%	75.26%
FHB + LCB	84.12%	81.44%	86.80%
FHB + NBB	82.37%	78.35%	86.39%
LCB + NBB	82.92%	79.31%	86.53%
FHB + LCB + NBB	85.09%	79.52%	90.65%

	DL with SRC		

FHB	75.74%	64.88%	86.60%
LCB	76.22%	62.75%	89.69%
NBB	78.63%	68.45%	88.80%
FHB + LCB	76.77%	66.39%	87.15%
FHB + NBB	82.54%	73.13%	91.96%
LCB + NBB	82.85%	73.47%	92.23%
FHB + LCB + NBB	83.54%	76.49%	90.58%

	ProCRC		

FHB	83.57%	73.61%	93.54%
LCB	84.30%	73.75%	94.85%
NBB	78.08%	63.71%	92.44%
FHB + LCB	87.11%	82.06%	92.16%
FHB + NBB	85.74%	79.73%	91.75%
LCB + NBB	85.43%	78.42%	92.44%
FHB + LCB + NBB	88.01%	84.95%	91.07%

	CRC		

FHB	78.76%	59.04%	98.49%
LCB	78.97%	59.86%	98.08%
NBB	76.19%	59.86%	92.51%
FHB + LCB	82.51%	67.42%	97.59%
FHB + NBB	82.65%	69.48%	95.81%
LCB + NBB	81.68%	68.45%	94.91%
FHB + LCB + NBB	84.43%	72.23%	96.63%

	Softmax		

FHB	83.68%	90.09%	79.15%
LCB	84.30%	91.48%	79.40%
NBB	78.97%	85.71%	74.48%
FHB + LCB	87.39%	91.14%	84.41%
FHB + NBB	85.15%	89.25%	81.92%
LCB + NBB	84.88%	89.67%	81.22%
FHB + LCB + NBB	87.39%	90.30%	85.00%

	Decision Tree		

FHB	78.21%	76.63%	79.79%
LCB	79.97%	78.76%	81.17%
NBB	73.61%	72.65%	74.57%
FHB + LCB	83.30%	81.44%	85.15%
FHB + NBB	80.86%	78.56%	83.16%
LCB + NBB	81.65%	79.79%	83.51%
FHB + LCB + NBB	81.58%	80.34%	82.82%

	AdaBoost		

FHB	83.40%	76.29%	90.52%
LCB	83.81%	76.70%	90.93%
NBB	79.97%	74.23%	85.70%
FHB + LCB	86.53%	84.81%	88.25%
FHB + NBB	84.40%	81.92%	86.87%
LCB + NBB	84.57%	80.27%	88.87%
FHB + LCB + NBB	86.56%	84.60%	88.52%

	LogitBoost		

FHB	83.40%	76.29%	90.52%
LCB	84.40%	79.59%	89.21%
NBB	79.90%	74.23%	85.57%
FHB + LCB	87.08%	84.47%	89.69%
FHB + NBB	84.78%	83.92%	85.64%
LCB + NBB	84.91%	81.10%	88.73%
FHB + LCB + NBB	87.70%	85.29%	90.10%

	Gentle Boost		

FHB	83.26%	76.43%	90.10%
LCB	83.81%	79.52%	88.11%
NBB	79.97%	73.75%	86.19%
FHB + LCB	87.04%	85.02%	89.07%
FHB + NBB	84.67%	84.12%	85.22%
LCB + NBB	85.02%	81.37%	88.66%
FHB + LCB + NBB	87.08%	84.26%	89.90%

## References

[1] McCullough P. A. (2007). Coronary artery disease. *Clinical Journal of the American Society of Nephrology*.

[2] T. H. Foundation What is heart disease?. https://www.theheartfoundation.org/heart-disease-facts/about-heart-disease/.

[3] W. H. Organization The top 10 causes of death. http://www.who.int/mediacentre/factsheets/fs310/en/.

[4] W. H. Organization Cardiovascular diseases (cvds). http://www.who.int/mediacentre/factsheets/fs317/en/.

[5] M. Clinic Heart disease - tests and diagnosis. http://www.mayoclinic.org/diseases-conditions/heart-disease/basics/tests-diagnosis/con-20034056.

[6] Davie A. P., Francis C. M., Love M. P. (1996). Value of the electrocardiogram in identifying heart failure due to left ventricular systolic dysfunction. *British Medical Journal*.

[7] Kuchar D. L., Thorburn C. W., Sammel N. L. (1987). Prediction of serious arrhythmic events after myocardial infarction: Signal-averaged electrocardiogram, Holter monitoring and radionuclide ventriculography. *Journal of the American College of Cardiology*.

[8] Leung D. Y., Davidson P. M., Cranney G. B., Walsh W. F. (1997). Thromboembolic risks of left atrial thrombus detected by transesophageal echocardiogram. *American Journal of Cardiology*.

[9] Wyman R. M., Safian R. D., Portway V., Skillman J. J., McKay R. G., Baim D. S. (1988). Current complications of diagnostic and therapeutic cardiac catheterization. *Journal of the American College of Cardiology*.

[10] Brenner D. J., Hall E. J. (2007). Computed tomography, an increasing source of radiation exposure. *N Engl J Med*.

[11] Nandalur K. R., Dwamena B. A., Choudhri A. F., Nandalur M. R., Carlos R. C. (2007). Diagnostic performance of stress cardiac magnetic resonance imaging in the detection of coronary artery disease: a meta-analysis. *Journal of the American College of Cardiology*.

[12] M. Clinic Boold tests for heart disease. http://www.mayoclinic.org/diseases-conditions/heart-disease/in-depth/heart-disease/art-20049357.

[13] Kim B. H., Lee S. H., Cho D. U., Oh S. Y. A proposal of heart diseases diagnosis method using analysis of face color.

[14] Zhang B., Kumar B. V. K. V., Zhang D. (2014). Noninvasive diabetes mellitus detection using facial block color with a sparse representation classifier. *IEEE Transactions on Biomedical Engineering*.

[15] Cai S., Zhang L., Zuo W., Feng X. A probabilistic collaborative representation based approach for pattern classification.

[16] Zhang L., Yang M., Feng X. Sparse representation or collaborative representation: Which helps face recognition?.

[17] Wright J., Yang A. Y., Ganesh A., Sastry S. S., Ma Y. (2009). Robust face recognition via sparse representation. *IEEE Transactions on Pattern Analysis and Machine Intelligence*.

[18] Wang X., Zhang D. (2010). An optimized tongue image color correction scheme. *IEEE Transactions on Information Technology in Biomedicine*.

[19] Wang H. (1999). *Huangdi Neijing*.

[20] Bing Z., Hongcai W. (2010). Basic theories of traditional Chinese medicine. *Singing Dragon*.

[21] Bing Z., Hongcai W. (2010). Diagnostics of traditional Chinese medicine. *Singing Dragon*.

[22] Youn S.-W., Park E.-S., Lee D.-H., Huh C.-H., Park K.-C. (2005). Does facial sebum excretion really affect the development of acne?. *British Journal of Dermatology*.

[23] Liu M., Guo Z. (2007). Hepatitis diagnosis using facial color image. *Medical Biometrics*.

[24] Zhang H., Patel V. M. (2017). Sparse Representation-Based Open Set Recognition. *IEEE Transactions on Pattern Analysis and Machine Intelligence*.

[25] Agarwal S., Roth D. (2002). Learning a Sparse Representation for Object Detection. *Computer Vision — ECCV 2002*.

[26] Mairal J., Elad M., Sapiro G. (2008). Sparse representation for color image restoration. *IEEE Transactions on Image Processing*.

[27] Lu T., Li S., Fang L., Ma Y., Benediktsson J. A. (2016). Spectral-Spatial Adaptive Sparse Representation for Hyperspectral Image Denoising. *IEEE Transactions on Geoscience and Remote Sensing*.

[28] Kang L., Yu C., Lin C., Yeh C. (2016). Image and Video Restoration and Enhancement via Sparse Representation. *Emerging Technologies in Intelligent Applications for Image and Video Processing*.

[29] Dong W., Fu F., Shi G. (2016). Hyperspectral image super-resolution via non-negative structured sparse representation. *IEEE Transactions on Image Processing*.

[30] Jain A. K., Duin R. P. W., Mao J. (2000). Statistical pattern recognition: a review. *IEEE Transactions on Pattern Analysis and Machine Intelligence*.

[31] Duda R. O., Hart P. E., Stork D. G. (2012). *Pattern classification*.

[32] Kreutz-Delgado K., Murray J. F., Rao B. D., Engan K., Lee T.-W., Sejnowski T. J. (2003). Dictionary learning algorithms for sparse representation. *Neural Computation*.

[33] Memisevic R., Zach C., Hinton G., Pollefeys M. Gated softmax classification.

[34] Brodley C. E., Friedl M. A. (1997). Decision tree classification of land cover from remotely sensed data. *Remote Sensing of Environment*.

[35] Khammari A., Nashashibi F., Abramson Y., Laurgeau C. Vehicle detection combining gradient analysis and AdaBoost classification.

[36] Cai Y.-D., Feng K.-Y., Lu W.-C., Chou K.-C. (2006). Using LogitBoost classifier to predict protein structural classes. *Journal of Theoretical Biology*.

[37] Lipowezky U. Indoor-outdoor detector for mobile phone cameras using gentle boosting.

[38] Glaros A. G., Kline R. B. (1988). Understanding the accuracy of tests with cutting scores: The sensitivity, specificity, and predictive value model. *Journal of Clinical Psychology*.

